# Metformin Directly Binds to MMP-9 to Improve Plaque Stability

**DOI:** 10.3390/jcdd10020054

**Published:** 2023-01-30

**Authors:** Xianda Chen, Shuaixing Wang, Wenli Xu, Mingming Zhao, Youyi Zhang, Han Xiao

**Affiliations:** 1 Department of Cardiology, Institute of Vascular Medicine, Peking University Third Hospital, Beijing 100191, China; 2 NHC Key Laboratory of Cardiovascular Molecular Biology and Regulatory Peptides, Beijing 100191, China; 3 Key Laboratory of Molecular Cardiovascular Science, Ministry of Education, Beijing 100191, China; 4 Beijing Key Laboratory of Cardiovascular Receptors Research, Beijing 100191, China; 5 Research Unit of Medical Science Research Management/Basic and Clinical Research of Metabolic Cardiovascular Diseases, Chinese Academy of Medical Sciences, Beijing 100191, China

**Keywords:** surface plasmon resonance, metformin, matrix metalloproteinase-9, plaque instability, atherosclerosis

## Abstract

Vulnerable atherosclerotic plaque rupture is the principal mechanism that accounts for myocardial infarction and stroke. High matrix metalloproteinase-9 (MMP-9) expression and activity have been proven to lead to plaque instability. Metformin, a first-line treatment for type 2 diabetes, is beneficial to plaque vulnerability. However, the mechanism underlying its anti-atherogenic effect remains unclear. Molecular docking and surface plasmon resonance experiments showed that metformin directly interacts with MMP-9, and incubated MMP-9 overexpressing HEK293A cells with metformin (1 μmol·L^−1^) significantly attenuates MMP-9’s activity using zymography and MMP activity assays. Moreover, metformin treatment drives MMP-9 degradation. Next, we constructed a carotid artery atherosclerotic plaque model and administered consecutive 14-day metformin (200 mg·kg^−1^·d^−1^) treatment by intragastric gavage. Immunofluorescence staining of the right carotid common artery and serum MMP activity assay results showed that metformin treatment decreased local plaque MMP-9 protein level and circulating MMP-9 activity, respectively. Histochemical staining revealed that after metformin treatment, the collagen content in plaque was significantly preserved, and the plaque vulnerability index decreased. These findings suggested that metformin improved atherosclerotic plaque stability by directly binding to MMP-9 and driving its degradation.

## 1. Introduction

Nearly 17.5 million people die each year from atherosclerosis-related diseases (31% of the global mortality). Of these, approximately 7.4 million died from coronary heart disease and 6.7 million from stroke [1]. Vulnerable atherosclerotic plaque rupture is the principal mechanism that accounts for myocardial infarction and stroke [2]. Therefore, there is a clinical need for plaque stabilization drugs.

Metformin, a biguanide, is the top choice of oral agent for the treatment of type 2 diabetes owing to its glucose-lowering effectiveness, safety, favorable effect on body weight, and low cost [3]. Moreover, metformin has been associated with decreased all-cause mortality and a reduced incidence of cardiovascular disease among patients with diabetes. A clinical trial investigated the effect of long-term metformin use and lifestyle at a diabetes prevention program and found that metformin was protective against atherosclerotic vascular disease early in diabetes development and potentially extended the range of this action to include high-risk male prediabetic subjects [4]. A recent meta-analysis showed the association between metformin and decreased cardiovascular mortality (95% CI, OR 0.44 [0.34−0.57]) or incidence of cardiovascular diseases (95% CI, OR 0.73 [0.59−0.90]) among patients with diabetes [5]. However, the molecular mechanism by which metformin improves atherosclerosis plaque stability remains unclear.

Vulnerable plaques are characterized by fragile, thin fibrous caps, massive lipid cores, intraplaque hemorrhage, immune activation, and increased levels of pro-inflammatory mediators (cytokines, chemokines, and matrix metalloproteinases) [6]. IL-6 stimulates the expression of adhesive molecules and results in an increase in the production and reactivity of acute phase indicators, such as C-reactive protein and TNF-α [7,8]. IL-18 and TNF-α are crucial for atherosclerotic plaque development and stability [9,10]. All of the cytokines mentioned above play a significant role in the formation and destabilization of atherosclerotic plaques. Mature plaques mainly comprise endothelial cells, vascular smooth muscle cells, macrophages, and fibrous caps containing extracellular matrix (ECM) components [11]. Among these components, the ECM is especially important for plaque stability [12]. Proteases have been implicated in the development and progression of atherosclerosis due to their ability to cause focal destruction of the ECM of blood vessels. Matrix metalloproteinase (MMP)-9, also known as gelatinase B, is a widely studied member of the MMP family. Histopathological studies have shown that MMP-9 is mainly distributed in the shoulder area, necrotic core, and fibrous cap area of atherosclerotic plaques, and the level and activity of MMP-9 in unstable plaques are higher than those in stable plaques [13,14,15]. Moreover, many studies have shown that high MMP-9 expression can be used as a predictor of atherosclerotic plaque instability, whereas its overexpression may lead to plaque instability [16,17,18]. Therefore, MMP-9 is a potential target for improving atherosclerotic plaque stability. However, whether metformin can target MMP-9 and inhibit its activity to stabilize plaque remains unclear.

Here, we report a novel mechanism by which metformin directly binds to MMP-9 and inhibits its activity to improve atherosclerotic plaque stability.

## 2. Materials and Methods

### 2.1. Mice

The investigations conformed to the US National Institutes of Health Guide for the Care and Use of Laboratory Animals (NIH Publication No. 85-23, revised 1996). Animal experiments were approved by the Committee of Peking University on Ethics of Animal Experiments (LA 2018-112) and conducted in accordance with the Guidelines for Animal Experiments, Peking University Health Science Center. Male ApoE knockout mice (ApoE-/-, C57BL/6J background) were purchased from Cyagen Biosciences Inc. (Suzhou, China) and used for the experiments. From 8 weeks of age, ApoE-/- mice were fed a high-fat, high-cholesterol diet containing 40 kcal% fat and 1.25% cholesterol (D12108C; Research Diets, New Brunswick, NJ, USA) for 14 weeks. All mice were housed in a specific pathogen-free environment under a 12 h/12 h light-dark cycle.

### 2.2. Carotid Collar Placement and Drug Treatment

Male ApoE knockout mice (8 weeks of age, C57BL/6J background) were fed a high-fat diet containing 40 kcal% fat and 1.25% cholesterol (D12108C; Research Diets, New Brunswick, NJ, USA) for 2 weeks. Carotid collar placement was performed 2 weeks later, and the operation process is briefly described as follows [19]: the mice were weighed, anesthetized by an intraperitoneal injection of 2% pentobarbital sodium (50 mg·kg^−1^), and their limbs were fixed on a thermostatic operating table. Erythromycin eye ointment was applied to the eyes of the mice to prevent dry eyes. Hair removal ointment was applied to remove neck and chest fur and fully expose the neck and chest surgical field. The epidermis was cut off at the median line of the neck using scissors, the right common carotid artery (RCCA) was bluntly separated with forceps, and the accompanying nerves and vessels were not damaged. A silicone collar with an inner diameter of about 0.3 mm (≈30% stenosis) was placed on the lateral side of the RCCA. The collar was fixed with a 6-0 silk thread and sutured for disinfection. Meloxicam (1.5 mg·kg^−1^) was injected intraperitoneally for analgesia after surgery and resuscitated on heat mats. High-fat feeding was continued for more than 3 months until plaque formation. Subsequently, metformin (Sigma-Aldrich, St. Louis, MO, USA; 200 mg·kg^−1^ body weight) or saline was administered by intragastric gavage for 14 consecutive days.

### 2.3. Histopathology and Immunofluorescence

The RCCAs from mice were harvested and embedded in an OCT compound (Lot# 4583; Tissue-Tek, USA). The OCT-embedded vascular tissue was sequentially sliced into slices approximately 6–8 μm thick using a microtome (Leica, Wetzlar, Germany), and placed on polylysine-coated glass slides. For all subsequent pathological staining (including immunofluorescence, oil red O, and Sirius red), 2–4 frozen sections of each vascular tissue with an interval of more than 50 μm were stained, and the average of the statistical values from the same sample was used as the final result [20].

To analyze plaque stability, serial sections (8 μm thick) were stained with picrosirius red to detect collagen deposition and oil red O to detect lipid deposition; both stains were analyzed by quantifying the positive area per total plaque area. Slices were incubated with primary antibodies against the macrophage marker CD-68 (1:50 dilution; Abcam, ab53444, Cambridge, UK) and smooth muscle cell marker α-SMA (1:50 dilution; Abcam, ab124964, Cambridge, UK), followed by incubation with fluorescence-conjugated secondary antibodies. The sections were mounted with 4’, 6-diamidino-2-phenylindole (DAPI; Abcam, ab104139, Cambridge, UK) for nuclei visualization.

To further characterize the carotid arteries, slices were incubated with the following primary antibodies: anti-MMP-9 (1:50 dilution; Invitrogen, MA5-15886, Carlsbad, CA, USA), anti-active MMP-9 (1:50 dilution; NOVUS, NBP2-13173, Carlsbad, CA, USA), anti-MMP-2 (1:50 dilution; Abcam, ab92536, Cambridge, UK), anti-MMP-12 (1:50 dilution; Proteintech, 22989-1-AP, Rosemont, IL, USA), anti-IL-1β (1:50 dilution; Bioss, bs0812R, Peking, China), anti-IL-6 (1:50 dilution; Proteintech, 66146-1-Ig, Rosemont, IL, USA), and anti-TNF-α (1:50 dilution; Abcam, ab1793, Cambridge, UK), followed by incubation with fluorescence-conjugated secondary antibodies. The sections were mounted with 4’, 6-diamidino-2-phenylindole (DAPI; Abcam, ab104139, Cambridge, UK) for nuclei visualization.

### 2.4. Western Blotting

Liver tissues and cell lines were lysed in a RIPA lysis buffer containing 1 mmol·L^−1^ phenylmethanesulfonyl fluoride (Beyotime Institute of Biotechnology, Beijing, China) at 4 °C for 30 min. The lysates were then centrifuged at 15,000× *g* for 10 min at 4 °C and their protein concentrations were determined using the BCA Protein Assay (Beyotime Institute of Biotechnology, Beijing, China). Samples were mixed with 5× SDS loading buffer, boiled for 5 min, and 50 μg of total protein was subjected to SDS-PAGE in 10% gels and transferred to nitrocellulose membranes. After blocking, the membranes were incubated overnight at 4 °C with the following primary antibodies: anti-MMP-9 (1:1000 dilution; Invitrogen, MA5-15886, Carlsbad, CA, USA), anti-p-AMPK (1:1000 dilution; CST, #2535, Danvers, MA, USA), anti-AMPK (1:1000 dilution; CST, #2532, Danvers, MA, USA), and anti-GAPDH (1:5000; CST, #2118, Danvers, MA, USA). The membranes were washed with Tris-buffered saline/0.1% Tween 20 (TBST) and incubated with secondary antibodies for 1 h at 25 °C. Signals were detected using Pierce™ ECL Western Blotting Substrate (Thermo Fisher Scientific, Waltham, MA, USA). Protein levels were quantified by calculating the grayscale value of each band using ImageJ (version 1.43, National Institutes of Health, Bethesda, MD, USA) software.

### 2.5. Matrix Metalloproteinases (MMPs) Activity Assay

Matrix metalloproteinases (MMPs) activity in mouse serum and cell culture supernatant was measured using Invitrogen DQ^TM^ luciferase-conjugated gelatin substrate (D12054; Invitrogen, Carlsbad, CA, USA), a fluorescent substrate that can detect protease activity with high sensitivity. The substrate consists of highly quenched fluorescein-labelled gelatin. After proteolytic digestion, the exhibited bright green fluorescence can be used to measure enzyme activity. Increased fluorescence intensity was monitored using a fluorescent microplate reader or fluorimeter. After receiving the cell supernatant, the cells were incubated with DQ gelatin, and a zinc-ion-containing buffer was added. After standing at room temperature and away from light for 24 h, the fluorescence intensity of each well was measured using a fluorescence microplate reader (TECAN, Männedorf, Switzerland).

### 2.6. Molecular Docking and Dynamics Simulation

The ligand metformin was processed using the Schrödinger 10.2 software (Schrödinger, LLC, NY, USA) LigPrep module. An OPLS3 force field was adopted for energy minimization. The crystal structure of MMP-9 was obtained from the RCSB Protein Data Bank. The crystallographic structure of 4WZV was prepared using the Protein Preparation Wizard module. A glide was applied to predict the potential binding mode of metformin with the MMP-9 protein. Following the docking results, an independent 50 ns molecular dynamics simulation was performed using Desmond. Na^+^ and Cl^−^ ions were each added at the physiological concentration of 0.15 mol·L^−1^ to ensure the overall neutrality of the systems. Simulations were conducted using an OPLS3 force field and a TIP3P explicit solvent model. The final size of the solvated system was approximately 20,000 atoms. A 5 ps recording interval was selected, and the NPT ensemble was employed with a fixed temperature of 300 K and pressure of 1.01 bar. The analysis tool of the simulation interactions diagram was used to monitor ligand–protein interactions.

### 2.7. Cell Culture, Plasmids, and Transfection

HEK 293A cells were obtained from the Cell Resource Center, Peking Union Medical College (which is the headquarter of National Science & Technology Infrastructure--National BioMedical Cell-Line Resource, NSTI-BMCR). Cells were maintained at 37 °C, with 5% CO_2_ in DMEM supplemented with 10% FBS and 10^4^ U∙mL^−1^ Pen/Strep. MMP-9 was overexpressed using an MMP-9-pcDNA3.1(+)-3Xflag plasmid synthesized by Ruibiotech (Beijing, China). Control plasmid did not contain sequences homologous to those of humans, mice, or rats. HEK 293A cells were seeded into 6-well plates (1.0 × 10^6^ cells/well) for 24 h and transfected with MMP-9 or control plasmid using lipofectamine 3000 (Invitrogen, Waltham, MA, USA) for 24 h, according to the manufacturer’s instructions. Furthermore, the transfected cells were incubated with metformin (Sigma-Aldrich, St. Louis, MO, USA; 1 μmol·L^−1^) for an additional 24 h. For the degradation experiment, the transfected HEK293A cells were pretreated with metformin for half an hour and incubated with cycloheximide (CHX; MedChemExpress, HY-12320; 10 μmol·L^−1^) to block protein synthesis for the indicated periods (0, 1, 2, 3 h). Lysates are harvested from the cells and analyzed by Western blotting.

### 2.8. Quantitative Real-Time PCR

Total RNA was extracted from the cell line using TRIzol reagent (Invitrogen, Carlsbad, CA, USA), according to the manufacturer’s protocol. Relative quantitation by real-time PCR was performed using SYBR Green to detect PCR products in real-time using the QuantStudioTM3 system (Applied Biosystems). A melting curve analysis was performed at the end of each PCR reaction. MMP-9 gene expression was expressed as a ratio to that of GAPDH, a housekeeping gene. Oligonucleotide primer sequences were as follows: Mmp-9, forward 5’-GGACCCGAAGCGGACATTG-3’ and reverse 5’-CGTCGTCGAAATGGGCATCT-3’; Gapdh, forward 5’-TGGATTTGGACGCATTGGTC-3’ and reverse 5’-TTTGCACTGGTACGTGTTGAT-3’.

### 2.9. Surface Plasmon Resonance (SPR) Spectroscopy

Experiments were performed at 25 °C using a Biacore T200, and the data were analyzed using Biacore T200 evaluation software 2.0 (GE Healthcare, Stockholm, Sweden). Human MMP-9 recombinant protein (911-MP; R&D Systems Incorporated, Minneapolis, MN, USA) was covalently coupled to a CM5 chip (GE Healthcare). All measurements were performed at 25 °C, using a TCNB buffer: 50 mmol·L^−1^ Tris, 10 mmol·L^−1^ CaCl_2_, 150 mmol·L^−1^ NaCl, 0.05% Brij-35 (*w*/*v*), and pH 7.5, and metformin was injected in a two-fold dilution concentration series (range, 0.0156–15.6 µmol·L^−1^). Steady-state values were calculated from the sensorgrams and plotted against concentrations. Data were fitted into a single-site binding model to calculate the KD value.

### 2.10. Zymography

Gelatinase activity was detected in HEK293A supernatants and recombinant human MMP-9 protein (911-MP; R&D Systems Incorporated, Minneapolis, MN, USA) after metformin incubation for 24 h. Zymography was performed according to the manufacturer’s instructions (Applygen, P1700, Beijing, China). Following electrophoresis, the gels were washed twice with 2.5% Triton X-100 to remove sodium dodecyl sulfate and further washed with 50 mmol·L^−1^ Tris–HCl pH 8.0. Gels were incubated for the following 20 h in an activation buffer (50 mmol·L^−1^ Tris–HCl supplemented with 5 mmol·L^−1^ CaCl_2_). The gels were stained with Coomassie brilliant blue R-250 and de-stained with 20% methanol and 10% acetic acid in distilled water until clear bands were visualized.

### 2.11. Statistics

Data are expressed as mean ± SD. All samples were independent, including those measured over time in the experiments. For parametric data, Student’s *t*-test or an analysis of variance (ANOVA) was used to analyze intergroup differences for normally distributed data. For parametric data with unequal variances, ANOVA with Tukey’s post hoc test was used. For non-parametric data, the Mann–Whitney U test with the exact method was used to analyze intergroup differences. A Kruskal–Wallis ANOVA combined with post hoc Tukey’s multiple comparison tests was performed when more than two groups were evaluated. Data were analyzed using GraphPad Prism software (version 8.0; GraphPad Software Inc., San Diego, CA, USA), and *p* < 0.05 was considered statistically significant.

## 3. Results

### 3.1. Matrix Metalloproteinase-9 (MMP-9) Is Predicted to Bind Directly to Metformin

**Figure 1:**
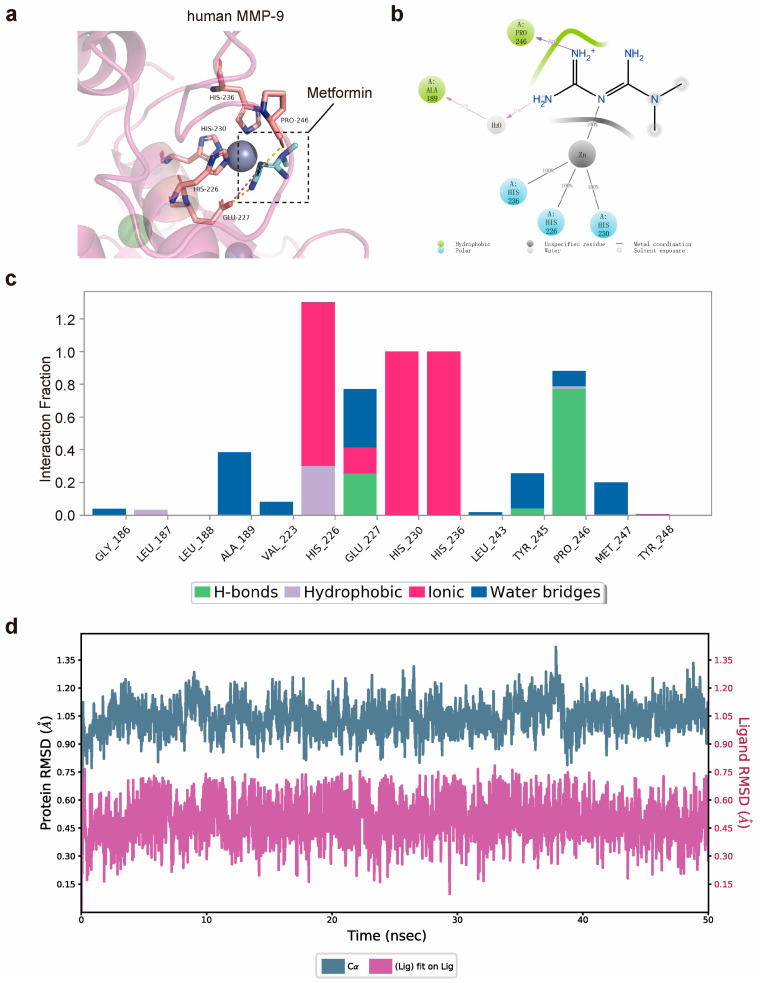
Matrix metalloproteinase-9 (MMP-9) is predicted to bind directly to metformin. (**a**) Predicted binding mode of metformin with MMP-9 (PDB id: 4wzv). (**b**,**c**) Protein–ligand contact histogram of metformin and the corresponding two-dimensional diagram predicted through MD simulations. A percentage value suggests that for X% of the simulation time, the specific interaction is maintained. (**d**) RMSD of the interaction between MMP-9 and the ligand metformin in MD simulations. MD, molecular dynamics; RMSD, root mean square deviation; MMP-9, matrix metalloproteinase-9.

We hypothesized that metformin inhibits MMP-9 activity through its direct interaction with MMP-9. Molecular modeling was performed to rationalize the activities of metformin against MMP-9. Metformin was situated in the active cavity, engaging in several interactions with MMP-9 (Figure 1a). Two hydrogen bonds were between the urea moiety and Pro-246 and Glu-227. Additionally, the protonated imine group formed an ionic bond with Glu-227. Notably, metal coordination was observed between metformin and the zinc ions, which might have strengthened the binding affinity. As shown in the protein–ligand contact histogram, the results were consistent with those of the docking study. The two hydrogen bonds formed by Pro-246 and Glu-227 were maintained at 76% and 30% of the simulation time, respectively (Figure 1b,c). A powerful coordination bond was formed between the nitrogen atom of metformin and the zinc metal ions. In addition, the amino group formed a hydrogen bond network through a water bridge with Ala-189. Further molecular dynamic (MD) simulation analysis revealed that the complex was stable during a 50 ns simulation (Figure 1d). Overall, these findings provided a better understanding of the metformin mechanisms and may facilitate a future search for optimized MMP-9 inhibitors.

### 3.2. Metformin Directly Interacts with MMP-9 and Attenuates Its Activity

**Figure 2:**
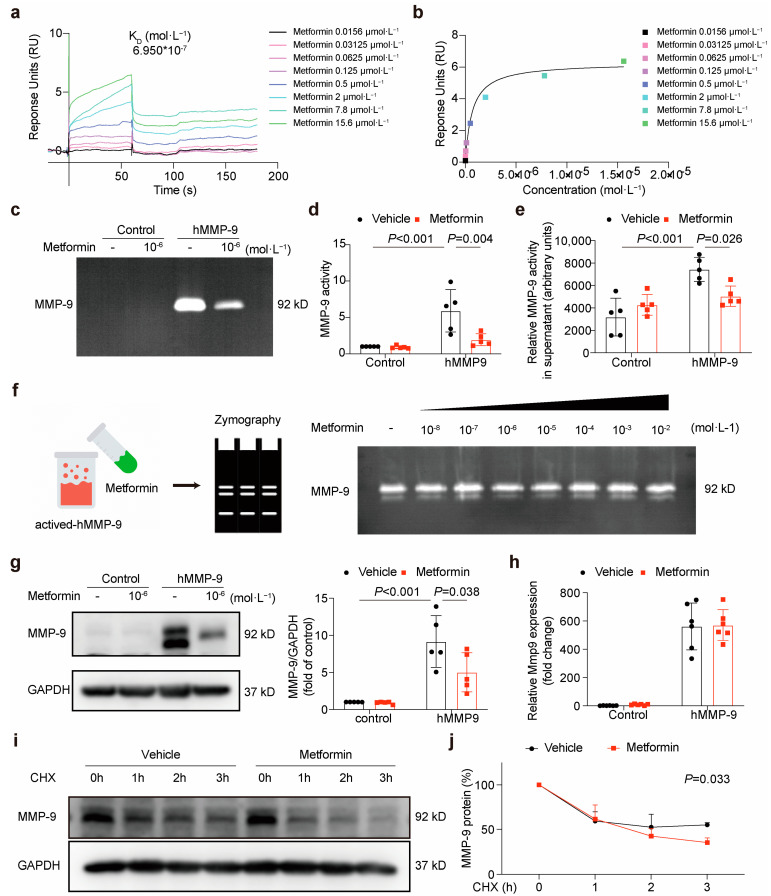
Metformin directly interacts with MMP-9 and attenuates its activity. (**a**,**b**) SPR analysis of the binding between metformin and MMP-9. Recombinant human MMP-9 protein was immobilized on an activated CM5 sensor chip, and metformin was then flowed across the chip. (**c**,**d**) Representative gelatin zymogram and the quantified values of the 92 kDa MMP-9 activity in the cultured supernatant. Data are shown as mean ± SD (two-way ANOVA followed by Tukey’s test, n = 5). (**e**) MMP activity in supernatant from cultured HEK293A cells was measured using a Gelatinase Assay Kit (two-way ANOVA followed by Tukey’s test, n = 5). (**f**) Representative gelatin zymogram of the recombinant human MMP-9 activity after incubation with different concentrations of metformin. (**g**) The exogenous MMP-9 protein level in HEK293A cells after incubation with metformin for 24 h (both of the 2 bands were quantified, two-way ANOVA followed by Tukey’s test, n = 5). (**h**) MMP-9 mRNA expression level in HEK293A cells after incubation with metformin for 24 h (n = 6). (**i**,**j**) Exogenous MMP-9 degradation in metformin-treated HEK293A cells when protein synthesis was inhibited by 10 μM cycloheximide (two-way ANOVA followed by Tukey’s test, n = 5).

To verify whether metformin directly binds to MMP-9, we conducted surface plasmon resonance (SPR) experiments. The findings of the SPR-based assay suggested that the binding of metformin to MMP-9 occurred with a KD of 0.6950 μmol·L−1 (Figure 2a,b). To examine the ability of metformin to inhibit MMP-9 activity, we constructed an overexpression plasmid for human MMP-9 and transfected it, or a control plasmid into HEK293A cells using lipofectamine (Figure S1). Next, we incubated the transfected cells with metformin (1 μmol·L^−1^) for 24 h, and the MMP-9 activity in the cultured supernatant was detected using zymography and an MMP activity assay. Both results indicated that metformin incubation significantly attenuated the activity of MMP-9 (Figure 2c–e).

To verify whether the inhibition of MMP-9 activity by metformin is a direct binding effect, we conducted a test tube experiment. The results showed that the activity of MMP-9 was not affected by metformin binding directly to MMP-9 (Figure 2f). However, Western blotting results suggested that metformin treatment could decrease the protein level of MMP-9 (Figure 2g). Western blot analysis of MMP-9 in the total cell lysate consistently revealed two bands of apparent molecular masses of 85 and 92 kDa. It was previously shown that the 85 kDa band represents an underglycosylated precursor form of MMP-9 found intracellularly, whereas the 92 kDa band represents a fully glycosylated mature form that is secreted into the extracellular space [21]. Further, we detected the transcription level of MMP-9 by polymerase chain reaction and found that metformin did not change the mRNA level of MMP-9 (Figure 2h).

Accordingly, we became interested in establishing whether metformin downregulated the MMP-9 protein level by driving its degradation. To this end, we used eukaryotic inhibitor cycloheximide to inhibit protein synthesis in HEK293A cells to study the degradation of MMP-9 with or without metformin. We found that the exogenous MMP-9 protein was continuously degraded from 1 to 3 h, and metformin treatment effectively decreased MMP-9 protein expression by accelerating its degradation (Figure 2i,j).

### 3.3. Metformin Inhibits Local Plaque and Circulation MMP-9 Activity in ApoE-/- Mice

**Figure 3:**
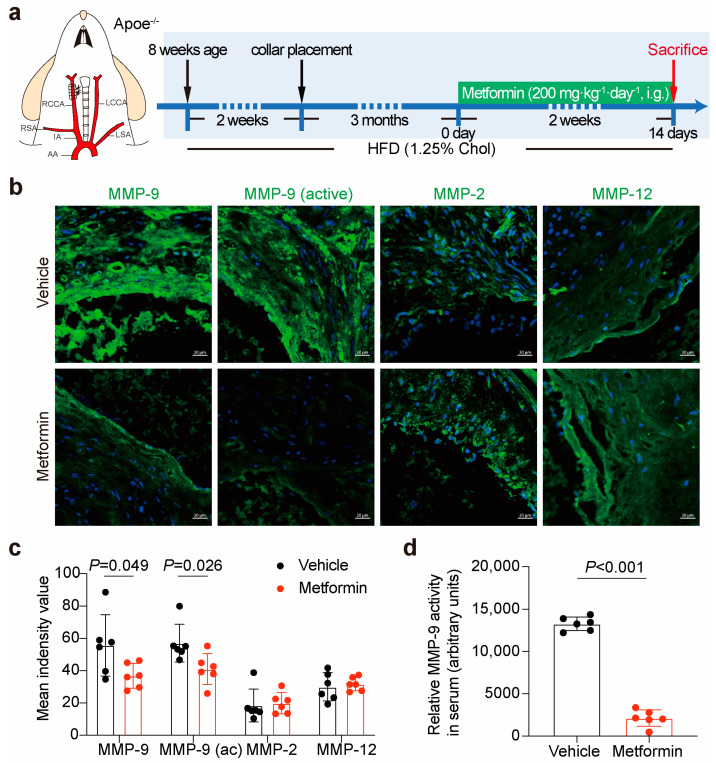
Metformin inhibits local plaque and circulating MMP-9 activity in ApoE-/- mice. (**a**) Flowchart illustrating the experimental procedure for actuating metformin treatment in a collar-induced carotid atherosclerotic plaque model. (**b**) Representative images of immunofluorescence staining for active-matrix metalloproteinase (MMP)-9, MMP-2, and MMP-12 in plaque after metformin treatment. Scale bars represent 20 μm. (**c**) Quantification of immunofluorescence staining for MMP family in plaque after metformin treatment. Unpaired Student’s *t*-test, n = 6 per group. (**d**) A Gelatinase Assay Kit was used to detect relative MMP activity in serum. Unpaired Student’s *t*-test, n = 6 per group.

To further confirm whether metformin inhibits MMP-9 activity in vivo, we constructed a carotid artery plaque model in ApoE-/- mice (Figures S2 and S3) [19]. After a consecutive 14-day metformin treatment (200 mg·kg^−1^) by intragastric gavage (Figure 3a and Figure S4), we found that active MMP-9 and MMP-9 expression decreased in the plaque by immunofluorescence staining. However, metformin did not affect MMP-2/12 expression, which was reported to be related to plaque instability (Figure 3b,c). Moreover, the serum MMP-9 activity was detected using an MMP activity assay (Figure 3d). The results showed that metformin treatment inhibited local plaque and circulating MMP-9 activity.

### 3.4. Metformin Improves Atherosclerotic Plaque Stability in ApoE-/- Mice

**Figure 4:**
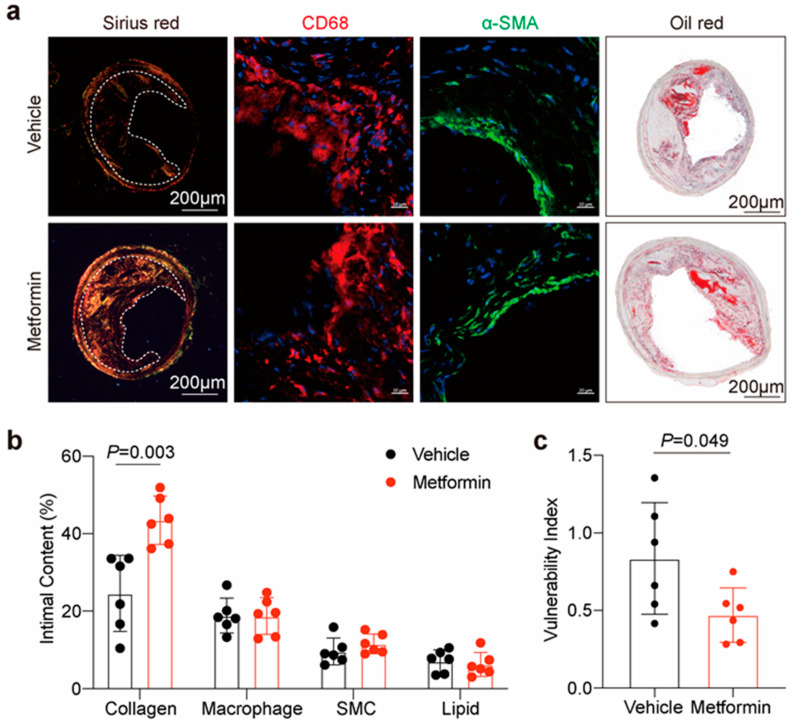
Metformin improves atherosclerotic plaque stability in ApoE-/- mice. (**a**) Representative images of Sirius red staining for plaque collagen, immunostaining for the macrophage marker CD-68, smooth muscle cell marker α-SMA, and oil red O staining for intimal lipid in plaque within the right common carotid artery. Scale bars for Sirius red staining and oil red O staining represent 200 μm and 20 μm for immunostaining. (**b**) Quantification of the positive area as a percentage of the whole plaque area. Unpaired Student’s *t*-test, n = 6 per group. (**c**) The vulnerability index is calculated by dividing the area of macrophage+lipid by that of smooth muscle cells+collagen. Unpaired Student’s *t*-test, n = 6 per group. Data are presented as the mean ± SD. HFD, high-fat diet; Chol, cholesterol.

To determine the protective effects of metformin on atherosclerosis, we assessed the vulnerability index (VI) of the RCCA plaque using histology. The composition of plaques, including macrophages, collagen, lipids, and smooth muscle cells (SMCs) was demonstrated by CD-68, Sirius red staining, oil red O staining, and α-SMA immunostaining, respectively (Figure 4a,b). Sirius red staining results showed that the collagen content was preserved by the metformin treatment. Oil red O staining, α-SMA, and CD-68 immunofluorescence results suggested that there were no significant differences in lipid, SMCs, and macrophage content after metformin treatment. As each feature alone is insufficient for identifying high-risk plaques, the ratio between stable and unstable plaque components is often used to calculate the VI (macrophage content + lipid core content)/(SMC content + collagen content) in experimental studies [22]. The results showed that with the metformin treatment, plaque VI was significantly decreased, indicating that metformin had a beneficial effect on plaque stability (Figure 4c).

## 4. Discussion

**Figure 5:**
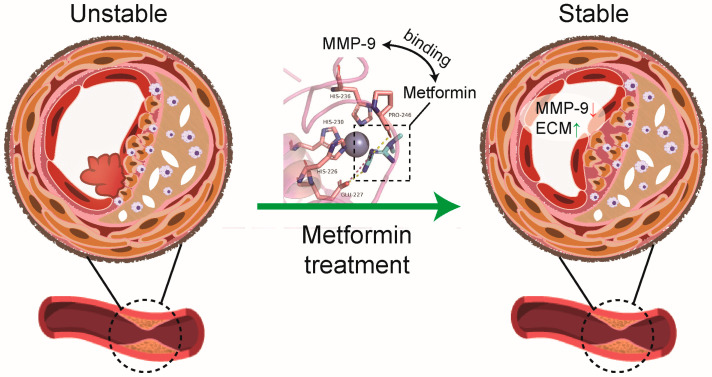
Schematic showing metformin directly binding to MMP-9 to improve plaque stability. MMP matrix metalloproteinase-9, ECM extracellular matrix.

In this study, we demonstrated that metformin directly binds to MMP-9 and accelerates its degradation. Furthermore, we proved that metformin improved atherosclerotic plaque stability by inhibiting local plaque and circulating MMP-9 in ApoE-/- mice (Figure 5). 

Collagens are most abundant in the extracellular matrix, joined by elastin that confers elastic recoil to the artery [23]. Loss of collagen, which normally provides the main tensile strength of the artery wall, is an important cause of atherosclerotic plaque rupture, which underlies most cases of ACS [24]. MMPs have specific proteolytic activity against the ECM, which can result in the thinning of the fibrous cap and plaque instability [11,25]. MMP-9, also known as gelatinase B, is a widely investigated member of the MMP family. Studies have shown a strong relationship between MMP-9 and plaque instability [26,27], which indicates that MMP-9 may be a therapeutic target for preventing plaque instability. Currently, inflammatory pathways are the main therapeutic targets for plaque instability, such as the monoclonal antibody inhibiting interleukin-1β (called canakinumab) [28] and PCSK-9 inhibitors [29]. Both canakinumab and PCSK-9 inhibitors have anti-inflammatory effects. Moreover, PCSK-9 inhibitors also have an inhibitory effect on MMP-2, but cannot inhibit MMP-9 [30]. So, the mechanism by which canakinumab and PCSK-9 inhibitors stabilize plaques may be different from metformin. In addition, there are few plaque-stabilizing drugs targeting MMP-9. Metformin interferes with the pathophysiology of multiple cancers and diabetes by reducing MMP-9 expression [31,32,33]. However, there are still many studies showing that metformin can increase MMP-9 expression [34], including some clinical trials [35,36]. Whether metformin stabilizes plaque by modulating MMP-9 activity and expression remains unknown. Our results indicated that metformin directly binds to MMP-9, and significantly downregulated MMP-9 expression/activity levels in local plaque and circulation, which may explain the role of metformin in improving plaque stability.

It is generally accepted that metformin inhibits pro-inflammatory cytokine release, such as IL-1β, IL-6, and TNF-α, to have anti-inflammatory effects [37,38,39,40,41]. Destabilization of the atherosclerotic plaque is associated with increased inflammatory cytokine production [42,43]. To investigate whether metformin protects plaque stability by inhibiting inflammation, we measured plaque IL-1β, IL-6, and TNF-α levels. The immunofluorescence staining results suggested that metformin treatment did not affect the levels of IL-1β, IL-6, and TNF-α in plaque (Figure S5). H. Wu et al. found that macrophage infiltration was significantly reduced after 16 weeks of metformin treatment [44]. However, our immunofluorescence staining results suggested that as short as two weeks of metformin treatment had no significant anti-inflammatory effect. This may have been due to the short treatment time in our animal model. Additionally, metformin has been reported to promote macrophage cholesterol efflux, thus decreasing the lipid content of atherosclerotic plaques and increasing plaque stability [44]. In this study, after consecutive 14-day metformin treatment (200 mg·kg^−1^) by intragastric gavage, we found that only the collagen content of the plaque was preserved, whereas intimal lipids, macrophages, and SMCs showed no significant difference, indicating that metformin improved plaque stability by reducing ECM degradation.

Metformin has protective effects by activating AMPK in intact cells and in vivo [45]. AMPK confers benefits in chronic inflammatory diseases, such as atherosclerosis, independent of its ability to normalize blood glucose levels. There was evidence that metformin inhibited TNF-α-induced MMP-9 upregulation in neutrophils, which might have been mediated via an AMPK-dependent pathway [46]. Metformin administration suppressed MMP-9/MMP-2 and mTOR expression and increased Akt and AMPK expression, indicating that metformin reduced the expression of MMPs via the AMPK signaling pathway [47]. In this study, we first found that metformin binds to MMP-9. The MMP-9 binding regions of metformin are situated in the active cavity and engage in several interactions with MMP-9. Moreover, the combination of metformin and MMP-9 significantly accelerated MMP-9 protein degradation, which may also account for the effect of metformin downregulating MMP-9 expression level and improving plaque stability. 

Protein homeostasis is responsible for basic cellular functions, such as the regulation of the level of key enzymes and the removal of abnormal proteins [48]. Our results suggested that the combination of metformin and MMP-9 significantly accelerated MMP-9 protein degradation. Chang Y et al. reported that cells treated with MG-132, a proteasome inhibitor, exhibited a significant MMP-9 protein accumulation compared to its accumulation in the untreated controls, indicating that the degradation of the MMP-9 protein is in a proteasome-dependent manner. Moreover, SMURF1, an E3 ubiquitin ligase, binds MMP-9 to promote its degradation [49]. In this study, we first found that metformin binds to MMP-9. The MMP-9-binding regions of metformin are situated in the active cavity and engage in several interactions with MMP-9. Further, MMP-9 was shown to have two N-glycosylation sites, which seems to be important for MMP-9 protein structure stabilization and secretion, on asparagine residues at position 38 in the propeptide domain and in the catalytic domain at position 120 [50,51,52]. In subsequent research, we have two directions to further explore the potential mechanism of metformin regulation of MMP-9: (1) metformin affects the binding of MMP-9 to SMURF1, thus promoting MMP-9 ubiquitination and accelerating its degradation; (2) metformin affects the role of N-glycosylation in MMP-9 and decreases MMP-9 protein structure stabilization.

In conclusion, we have demonstrated that metformin directly binds to MMP-9 and accelerates its degradation, thus preserving the collagen content of plaque and improving atherosclerotic plaque stability. Further, these findings could significantly impact the development of the search for new drugs and pleiotropic mechanisms.

## Data Availability

The data presented in this study are available in this article.

## Supplementary Materials

The following supporting information can be downloaded at: https://www.mdpi.com/article/10.3390/jcdd10020054/s1, Figure S1: MMP-9 was successfully overexpressed in HEK293A cells. Figure S2: Serum triglyceride and total cholesterol levels increased in animal models. Figure S3: The carotid plaque model was successfully constructed. Figure S4: Metformin successfully activated AMPK. Figure S5: Metformin treatment had no significant anti-inflammatory effect in our model.
