# Supplementary material for: Metformin Directly Binds to MMP-9 to Improve Plaque Stability
Source: J Cardiovasc Dev Dis. 2023 Jan 30;10(2):54. doi: 10.3390/jcdd10020054 (PMC9962015; doi:10.3390/jcdd10020054)
Supplement: Supplementary file 1 [file jcdd-10-00054-s001.zip › jcdd-2146331-supplementary.pdf]

Supplementary file for

Metformin directly binds to MMP-9 to improve plaque stability

This file includes:

Supplementary figures S1 to S5

Supplementary figures and supplementary figure legends

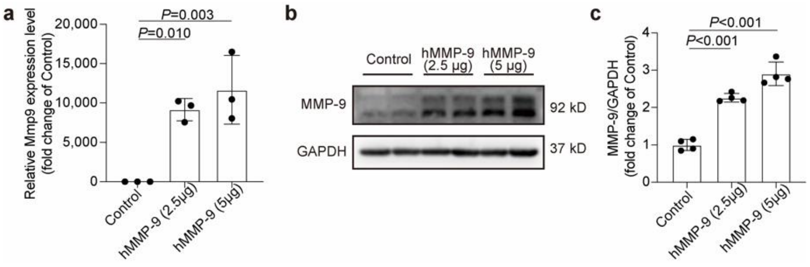

Figure S1. MMP-9 was successfully overexpressed in HEK293A cells. (a) Quantitative polymerase chain reaction (QPCR) for Mmp9 mRNA expression level of plasmid-transfected HEK293A cells. *n*=3; One-way ANOVA, Dunnett post hoc. Scatter plots show fold change of *Mmp9* gene expression level normalized to the Control group average. **(b)** Western blot showing the level of MMP-9 in plasmid-transfected HEK293A cells. **(c)** Quantification of band densities of Western blot. *n*=4; One-way ANOVA, Dunnett post hoc. Scatter plots show fold change of MMP-9 protein expression level normalized to the Control group average.

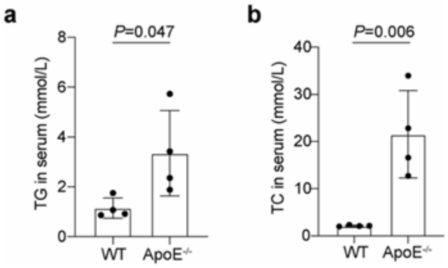

Figure S2. Serum triglyceride and total cholesterol levels increased in animal models. (a) Triglyceride content in the serum of Apoe knockout and wild-type mice. Scatter plots show the concentration of triglyceride in serum. **(b)** Serum total cholesterol in Apoe knockout mice and wild-type mice. Unpaired Student's t-test, *n*=4. Scatter plots show the concentration of total cholesterol in serum.

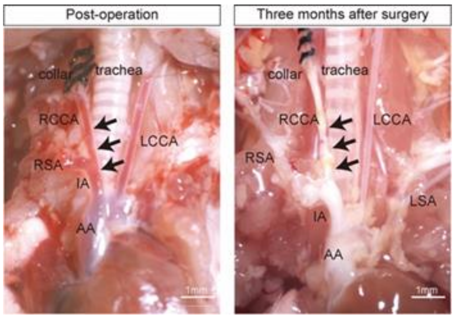

Figure S3. The carotid plaque model was successfully constructed. Anatomical diagram of the neck vessels of the post-operative model mice (left panel), showing smooth and transparent right common carotid artery (black arrow); Anatomical diagram of neck vessels in model mice 3 months after surgery (right panel). Diffuse, porcelain white continuous plaque formation can be seen throughout the right common carotid artery (black arrow), scale bar: 1mm. *RCCA right carotid common artery, LCCA left carotid common artery, RSA right subclavian artery, IA innominate artery, AA ascending aorta.*

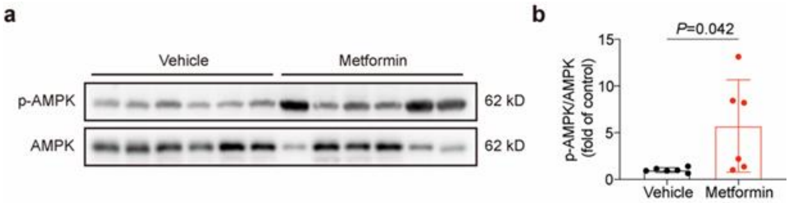

Figure S4. Metformin successfully activated AMPK. (a) Western blot showing the level of p-AMPK in plaque model mice livers after consecutive 14-day metformin treatment (200 mg·kg−1) by intragastric gavage. Total AMPK was used as a loading control. **(b)** Quantification of band densities of Western blot. Unpaired Student's t-test, n = 6 per group. *AMPK AMP-activated protein.* Scatter plots show the relative phosphorylation level of AMPK normalized to the Vehicle group average.

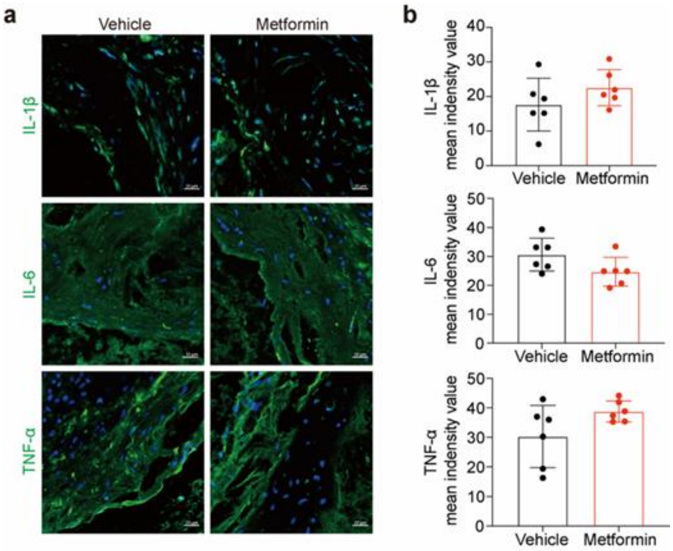

Figure S5. Metformin treatment had no significant anti-inflammatory effect in our model. (a,b) Representative images and quantification of immunofluorescence staining for inflammatory cytokines IL-1β, IL-6, and TNF-α in plaques. Scale bars represent 20 μm. Unpaired Student's

t-test, n = 6 per group. Scatter plots show the mean indensity values of the inflammatory cytokines IL-1β, IL-6, and TNF-α in plaques.
